# An Integrated Positioning and Attitude Determination System for Immersed Tunnel Elements: A Simulation Study

**DOI:** 10.3390/s20247296

**Published:** 2020-12-18

**Authors:** Guanqing Li, Lasse Klingbeil, Florian Zimmermann, Shengxiang Huang, Heiner Kuhlmann

**Affiliations:** 1 School of Geodesy and Geomatics, Wuhan University, Wuhan 430079, China; liguanqing@whu.edu.cn; 2 Institute of Geodesy and Geoinformation, University of Bonn, D-53115 Bonn, Germany; klingbeil@igg.uni-bonn.de (L.K.); zimmermann@igg.uni-bonn.de (F.Z.); heiner.kuhlmann@uni-bonn.de (H.K.)

**Keywords:** immersed tunnel element, positioning, attitude determination, GNSS, inclinometer, range-measurement

## Abstract

Immersed tunnel elements need to be exactly controlled during their immersion process. Position and attitude of the element should be determined quickly and accurately to navigate the element from the holding area to the final location in the tunnel trench. In this paper, a newly-developed positioning and attitude determination system, integrating a 3-antenna Global Navigation Satellite System (GNSS) system, an inclinometer and a range-measurement system, is presented. The system is designed to provide the absolute position of both ends of the element with sufficient accuracy in real time. Special attention in the accuracy analysis is paid to the influence of GNSS multipath error and sound speed profile. Simulations are conducted to illustrate the performance of the system in different scenarios. If both elements are very close, the accuracies of the system are higher than 0.02 m in the directions perpendicular to and along the tunnel axis.

## 1. Introduction

**Figure 1:**
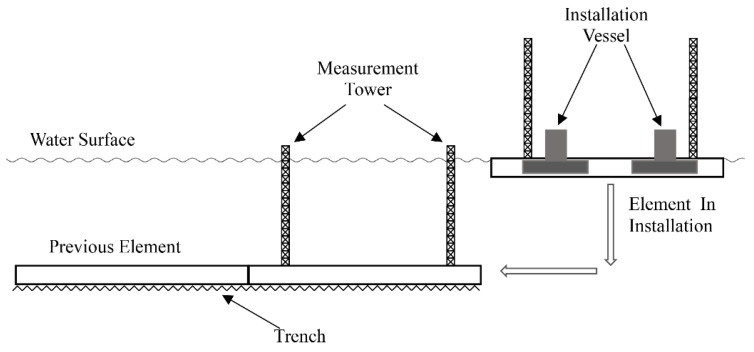
The installation of an immersed tunnel element.

An immersed tunnel consists of one or more elements that are prefabricated, floated to the site, installed one by one, and connected to the previous one in the tunnel trench under water [1]. The installation of an immersed tunnel element is shown in Figure 1. An immersed tunnel element is usually quite large, e.g., the outer dimension of the Øresund Link element is 8.5 m×41.7 m×176 m [2]; Busan Geoje Link tunnel element dimension is 9.8 m×26.5 m×180 m [3]; Hong Kong-Zhuhai-Macau (HZM) Link tunnel element dimension is 11.4 m×38 m×180 m [4]. Such large-sized prefabricated elements need to be exactly controlled during their immersion process. Position and attitude of the element should be determined quickly and accurately to navigate the element to the final position in the tunnel trench to ensure the water tightness of the immersion joints, the safety of the structure and the tunnel alignment. In general, the accuracy requirement of the immersed tunnel element installation in the direction perpendicular to the tunnel axis is ±0.035 m when the element is very close to the previous element. In the initial stage of the element installation, the accuracy can be released by a factor of 2.

**Figure 2:**
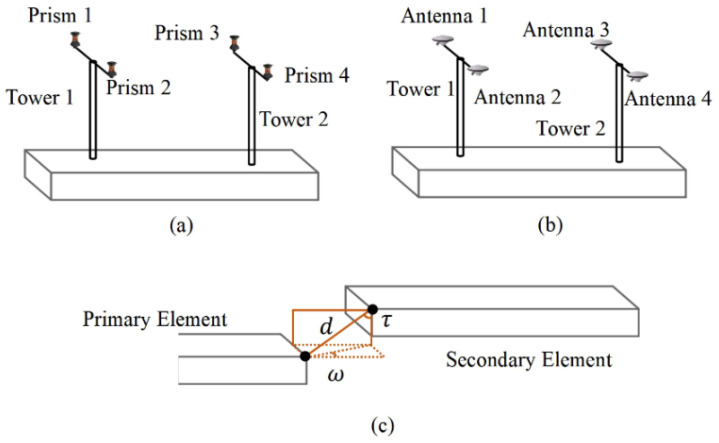
Present positioning and attitude determination methods of immersed tunnel element. (**a**) the measurement tower system with prisms, (**b**) the measurement tower system with GNSS antennas, (**c**) the taut-wire system.

The traditional positioning method for immersed tunnels is the measurement tower system. The measurement tower system consists of towers, which are fixed at the mating and free end of the element. Three total stations onshore are used to measure the positions of prisms fixed to the top of the tower prior to immersion, as shown in Figure 2a. The position and yaw of the element can be provided. But this method is inapplicable if the tunnel is more than 1 km from the shore. GNSS antennas can replace the prisms, as shown in Figure 2b. The relative position between prisms (or antennas) and the element is determined after the prefabrication. During element immersion, the positions of prisms (or antennas) are measured using total stations (or RTK, short for real time kinematic) in real time and the absolute position and attitude of the element can be calculated [5,6,7]. The maximum bottom depths of Øresund Link immersed tunnel, Busan Geoje Link tunnel, HZM link tunnel and Marmaray immersed tunnel are 30, 48, 50 and 60 m. The Marmaray immersed tunnel is currently the deepest immersed tunnel in the world. In order to avoid the influence of deformation of the measurement tower under deep water conditions, an acoustic system is designed [7,8,9,10]. The acoustic system consists of a transducer and several transponders. The transducer is mounted on the mating end of the element in installation (in the latter also called ‘secondary element’) and the transponders are mounted at known positions on the free end of the previous element (in the latter also called ‘primary element’). By measuring the sound travelling time between the two elements, the relative position of the element in installation with respect to the previous element can be calculated. If the distances between the element in installation and the previous one are measured in a mechanical way rather than by sound, it is called taut-wire system [5,8]. A taut-wire system includes distance sensors, cable units and other accessories. The relative position of the two elements can be obtained when the distance d and angles, τ and ω, are measured, as shown in Figure 2c.

The measurement tower system can determine the absolute position and attitude of the element, but it needs two towers. The acoustic and taut-wire system can only provide the position of the mating end relative to the primary element, and the position of the free end is calculated based on the element size and attitude. Recently, two or more different positioning systems have sometimes been adopted simultaneously [6,7,8]. The Guangzhou Zhoutouzui project adopted a measurement tower system with both total stations and prisms, as well as Global Position System (GPS) [7]. For the HZM link immersed tunnel construction, both a measurement tower system (with GPS) as well as an acoustic system were used [6]. All three methods, the measurement tower system (with total stations and prisms), the taut-wire system, and the sonar system were used in the Busan Geoje immersed tunnel [8]. Although multiple systems are usually used for element positioning, each system still works on its own. All systems except one are backups.

In recent years, the use of multiple GNSS antennas for positioning and attitude determination has extensively been researched, such as in airborne, shipborne and vehicle-borne applications [11,12,13,14,15,16]. To make the attitude determination a much more efficient and accurate method, many studies have focused on the instantaneous GNSS ambiguity resolution for multi-antennas [17,18,19,20]. Among them, the constrained LAMBDA method makes use of the given baseline length, works on a stronger model and achieves higher ambiguity success rates. These studies provide a solid foundation for the use of GNSS-based attitude determination in immersed tunnel application.

**Figure 3:**
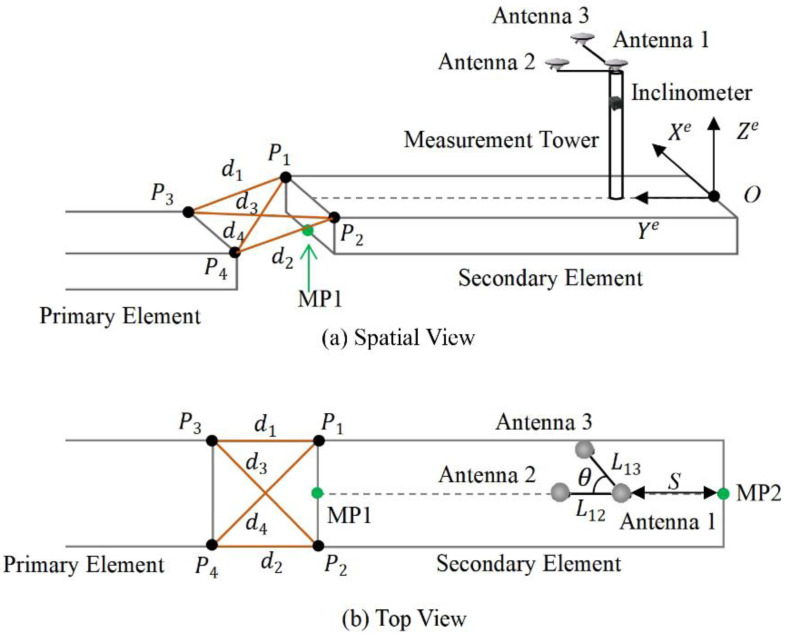
Element positioning attitude determination using an integrated system.

In order to reduce the number of measurement towers, and to obtain the absolute position of both ends of the element with sufficient accuracy in real time during the immersion process, a newly-developed integrated positioning and attitude determination system is presented. Two feature points, MP1 and MP2, are designed to indicate the element position (Figure 3). The two points are the bottom midpoints of the mating and free end of the element. MP1 is at the mating end. The vertical direction of the tunnel axis is designed to be parallel to the north direction, so the north positioning accuracy of the two points is the lateral accuracy. More attention should be paid to the accuracy of the north direction in this paper. The element attitude is defined as the orientation between the element fixed coordinate system and the navigation coordinate system.

The contribution of this paper is the development and simulation-based evaluation of the integrated positioning and attitude determination system of the element immersion. The rest of this article is arranged as follows. Firstly, the design of the integrated system and the algorithms are presented in detail, including the coordinate frames involved. Afterwards, the simulation errors, such as GNSS errors, roll and pitch angles errors, and distance errors are analyzed. Finally, Monte Carlo-based simulations are conducted to study the position and attitude accuracy performance of the system for different scenarios. The influence of different kinds of error of the observations on the element position is analyzed.

## 2. Integrated Positioning and Attitude Determination System of Element Immersion

### 2.1. Coordinate Systems

In order to define the element position and attitude precisely, four coordinate systems are used. These are (1) the Earth-Centered, Earth-Fixed (ECEF) Coordinate System (e.g., International Terrestrial Reference Frame, China Geodetic Coordinate System 2000 and WGS84, short for World Geodetic System 1984), (2) the navigation coordinate system, (3) the element-fixed coordinate system, and (4) a local plane coordinate and height system (realized by Gauss–Krüger or Universal Transverse Mercator projection). The four coordinate systems are shown in Table 1. In this paper, the ECEF Coordinate System refers to WGS84. The parameters of WGS84, navigation and element coordinate are distinguished by the superscript. The superscripts, w, n and e, denote the Earth frame, navigation and element-fixed coordinates, respectively. (N,E,H) represent the local coordinates.

The element fixed coordinate system is defined by the plane of the upper surface of the element, as shown in Figure 3. The yaw direction is the element’s center line ($Y^{e}$-axis) laying on the plane of element upper surface. The $X^{e}$-axis is perpendicular to the center line pointing to the right and laying on the plane of element upper surface. The $Z^{e}$-axis forms a right-handed coordinate system with $X^{e}$ and $Y^{e}$ axes. The attitude is defined as the orientation between the element fixed coordinate system and the navigation coordinate system. Roll (α) is the rotation angle around $Y^{e}$-axis, left-side up positive. Pitch (β) is the angle of rotation around $X^{e}$-axis, upward positive. Yaw (γ) is the rotation angle about $Z^{e}$-axis, clockwise positive.

### 2.2. Sensors System

The integrated system consists of a measurement tower with three GNSS antennas, an inclinometer and a range measurement system (acoustic system), as shown in Figure 3. Three antennas are mounted on the top of a measurement tower (access shaft) fixed on the element. Think antennas, measurement tower and element a rigid body. Antenna 1 is the main antenna and the baseline between antenna 1 and 2 is parallel to $Y^{e}$-axis. The coordinates of antenna 1, 2 and 3 in element fixed coordinate system are (0, S, h), $\left(0,\;S+L_{12},\;h\right)$ and $\left(L_{13}\sin\theta ,\;S+L_{13}\cos\theta ,\;h\right)$. S is the distance from the projection of antenna 1 on the plane of element upper surface to the free end; h is the perpendicular distance from antenna 1 to the plane of element upper surface; $L_{12}$ is the distance between antenna 1 and 2; $L_{13}$ is the distance between antenna 1 and 3; θ is the angle between baseline 1→2 and 1→3. The inclinometer has two measuring axes. It provides roll and pitch angle of the element after the inclinometer assembly error calibration. The range measurement system measures the four distances between the two upper corners of the mating end of the secondary element ($P_{1}$ and $P_{2}$) and the two upper corners of the free end of the primary element ($P_{3}$ and $P_{4}$). The measurement tower with GNSS antennas, the inclinometer and the range measurement system (acoustic system) have been applied and verified in multiple immersed tunnel projects, respectively. And they show good performances during the installation of the elements. Hence, we consider that the motion of the elements will have no significant impact on the integrated system. But this has yet to be tested in actual projects.

### 2.3. Estimation of Parameters

#### 2.3.1. Definition of Parameters

The coordinates of any point in the element can be calculated based on the position of antenna 1 and the attitude angles. Hence, in the integrated system six parameters are defined. The parameter vector X is ${\left(X_{1}^{w},\;Y_{1}^{w},\;Z_{1}^{w},\alpha ,\beta ,\gamma \right)}^{T}$, where:

- $\left(X_{1}^{w},\;Y_{1}^{w},\;Z_{1}^{w}\right)$: coordinate of antenna 1 in WGS84;
- (α,β,γ): roll, pitch and yaw angle of the element.

After obtaining the parameters vector, we can get the local coordinates of any point in the element by coordinate transformation. In this paper, the interested points in the element are MP1 and MP2.

#### 2.3.2. Functional Model of Positioning and Attitude Determination

The integrated system consists of 15 observations, including the position of antenna 1, two baselines, two attitude angles and four distances. The observation vector L is:

(1) $$L={\left[\begin{array}{ccccccccccccccc} x_{1}^{w} & y_{1}^{w} & z_{1}^{w} & x_{12}^{w} & y_{12}^{w} & z_{12}^{w} & x_{13}^{w} & y_{13}^{w} & z_{13}^{w} & d_{1} & d_{2} & d_{3} & d_{4} & \alpha_{L} & \beta_{L} \end{array}\right]}^{T},$$

To distinguish the observations from the parameters, observations are represented in lowercase letters and the attitude angles have a subscript *L*. The list of the observation vector is

- $\left(x_{1}^{w},\;y_{1}^{w},\;z_{1}^{w}\right)$: coordinate of antenna 1 in WGS84 measured by GNSS
- $\left(x_{12}^{w},\;y_{12}^{w},\;z_{12}^{w}\right)$: baseline 1→2 in WGS84 measured by GNSS
- $\left(x_{13}^{w},\;y_{13}^{w},\;z_{13}^{w}\right)$: baseline 1→3 in WGS84 measured by GNSS
- $\left(d_{1},d_{2},d_{3},d_{4}\right)$: spatial distances measured by the acoustic system
- $\left(\alpha_{L},\beta_{L}\right)$: roll and pitch angle of the element measured by the inclinometer

How to obtain the coordinate of antenna 1, baseline 1→2 and 1→3 based on GNSS observations is beyond the scope of this paper. We assume that we’ve got these values. For the position of antenna 1, the functional model is:

(2) $$L_{1}=\left[\begin{array}{c} x_{1}^{w}\\ y_{1}^{w}\\ z_{1}^{w} \end{array}\right]=\left[\begin{array}{cccccc} 1 & 0 & 0 & 0 & 0 & 0\\ 0 & 1 & 0 & 0 & 0 & 0\\ 0 & 0 & 1 & 0 & 0 & 0 \end{array}\right]X=A_{1}X,$$

Coordinates of antenna 1, 2 and 3 in element fixed coordinate system are known. According to coordinate transformation formula, the two baselines in WGS84, $s_{12}^{w}$ and $s_{13}^{w}$, can be expressed as:

(3) $$s_{12}^{w}=\left[\begin{array}{c} x_{12}^{w}\\ y_{12}^{w}\\ z_{12}^{w} \end{array}\right]={\left(R_{w}^{n}\right)}^{T}{\left(R_{n}^{e}\right)}^{T}s_{12}^{e}=f\left(X\right),$$

(4) $$s_{13}^{w}=\left[\begin{array}{c} x_{13}^{w}\\ y_{13}^{w}\\ z_{13}^{w} \end{array}\right]={\left(R_{w}^{n}\right)}^{T}{\left(R_{n}^{e}\right)}^{T}s_{13}^{e}=g\left(X\right),$$

where $R_{w}^{n}$ is the transformation matrix from WGS84 to navigation coordinate system. $R_{w}^{n}$ can be computed from the geographic coordinates of antenna 1, ($Lat_{1},Lon_{1},H_{1})$. The transformation matrix from navigation coordinate to element coordinate, $R_{n}^{e}$, can be computed from the attitude angles of the element, (α,β,γ).

Combine Equations (3) and (4), so the baseline functional model is:

(5) $$L_{2}=\left[\begin{array}{c} s_{12}^{w}\\ s_{13}^{w} \end{array}\right]=\left[\begin{array}{c} f\left(X\right)\\ g\left(X\right) \end{array}\right],$$

For the roll and pitch angles, the functional model is:

(6) $$L_{3}=\left[\begin{array}{c} \alpha_{L}\\ \beta_{L} \end{array}\right]=\left[\begin{array}{cccccc} 0 & 0 & 0 & 1 & 0 & 0\\ 0 & 0 & 0 & 0 & 1 & 0 \end{array}\right]X=A_{3}X,$$

The WGS84 coordinates of the two free end upper corners of the primary element ($P_{3}$ and $P_{4}$) are accurately measured by the final survey after the element installation. The accurate element coordinates of the two mating end upper corners of the secondary element ($P_{1}$ and $P_{2}$) are determined before the element is launched. According to coordinate transformation formula, WGS84 coordinates of $P_{1}$ and $P_{2}$ can be calculated as:

(7) $$\left[\begin{array}{c} X_{P_{1}}^{w}\\ Y_{P_{1}}^{w}\\ Z_{P_{1}}^{w} \end{array}\right]={\left(R_{w}^{n}\right)}^{T}{\left(R_{n}^{e}\right)}^{T}\left\{\left[\begin{array}{c} X_{P_{1}}^{e}\\ Y_{P_{1}}^{e}\\ Z_{P_{1}}^{e} \end{array}\right]-\left[\begin{array}{c} 0\\ S\\ h \end{array}\right]\right\}+\left[\begin{array}{c} X_{1}^{w}\\ Y_{1}^{w}\\ Z_{1}^{w} \end{array}\right],$$

(8) $$\left[\begin{array}{c} X_{P_{2}}^{w}\\ Y_{P_{2}}^{w}\\ Z_{P_{2}}^{w} \end{array}\right]={\left(R_{w}^{n}\right)}^{T}{\left(R_{n}^{e}\right)}^{T}\left\{\left[\begin{array}{c} X_{P_{2}}^{e}\\ Y_{P_{2}}^{e}\\ Z_{P_{2}}^{e} \end{array}\right]-\left[\begin{array}{c} 0\\ S\\ h \end{array}\right]\right\}+\left[\begin{array}{c} X_{1}^{w}\\ Y_{1}^{w}\\ Z_{1}^{w} \end{array}\right],$$

where ${\left(0,S,h\right)}^{T}$ is the element coordinate of antenna 1. For distances, the functional model is:

(9) $$L_{4}=[\begin{array}{c} d_{1}\\ d_{2}\\ d_{3}\\ d_{4} \end{array}]==[\begin{array}{c} \sqrt{{\left(X_{P_{1}}^{w}-X_{P_{3}}^{w}\right)}^{2}+{\left(Y_{P_{1}}^{w}-Y_{P_{3}}^{w}\right)}^{2}+{\left(Z_{P_{1}}^{w}-Z_{P_{3}}^{w}\right)}^{2}}\\ \sqrt{{\left(X_{P_{2}}^{w}-X_{P_{4}}^{w}\right)}^{2}+{\left(Y_{P_{2}}^{w}-Y_{P_{4}}^{w}\right)}^{2}+{\left(Z_{P_{2}}^{w}-Z_{P_{4}}^{w}\right)}^{2}}\\ \sqrt{{\left(X_{P_{2}}^{w}-X_{P_{3}}^{w}\right)}^{2}+{\left(Y_{P_{2}}^{w}-Y_{P_{3}}^{w}\right)}^{2}+{\left(Z_{P_{2}}^{w}-Z_{P_{3}}^{w}\right)}^{2}}\\ \sqrt{{\left(X_{P_{1}}^{w}-X_{P_{4}}^{w}\right)}^{2}+{\left(Y_{P_{1}}^{w}-Y_{P_{4}}^{w}\right)}^{2}+{\left(Z_{P_{1}}^{w}-Z_{P_{4}}^{w}\right)}^{2}} \end{array}]=\psi \left(X\right),$$

Hence, combine Formulas (2), (5), (6) and (9), the nonlinear functional model is established as:

(10) $$L_{15\times 1}=\left[\begin{array}{c} L_{1}\\ L_{2}\\ L_{3}\\ L_{4} \end{array}\right]=\left[\begin{array}{c} A_{1}X\\ f\left(X\right)\\ g\left(X\right)\\ A_{3}X\\ \psi \left(X\right) \end{array}\right]=h\left(X\right),$$

To linearize nonlinear functional models, we have:

(11) $$X=X^{0}+\tilde{x},$$

where $X^{0}$ is the approximate value of X and $\tilde{x}$ is the small value. In order to obtain the linearized functional models of different kinds of observation, Formulas (5) and (9) can be expanded to linear form:

(12) $$L_{2}=\left[\begin{array}{c} f\left(X^{0}\right)\\ g\left(X^{0}\right) \end{array}\right]+{\left[\begin{array}{c} \frac{\partial f}{\partial X}\\ \frac{\partial g}{\partial X} \end{array}\right]}_{X^{0}}\tilde{x}=\left[\begin{array}{c} f\left(X^{0}\right)\\ g\left(X^{0}\right) \end{array}\right]+A_{2}\tilde{x},$$

(13) $$L_{4}=\psi \left(X^{0}\right)+{\left[\frac{\partial \psi }{\partial X}\right]}_{X^{0}}\tilde{x}=\psi \left(X^{0}\right)+A_{4}\tilde{x},$$

Substitute Formula (11) into Formulas (2) and (6) so as to unify the forms of functional models:

(14) $$L_{1}=A_{1}X^{0}+A_{1}\tilde{x},$$

(15) $$L_{3}=A_{3}X^{0}+A_{3}\tilde{x},$$

Finally, the linear functional model of positioning and attitude determination is established:

(16) $$L_{15\times 1}=\left[\begin{array}{c} L_{1}\\ L_{2}\\ L_{3}\\ L_{4} \end{array}\right]=\left[\begin{array}{c} A_{1}\\ A_{2}\\ A_{3}\\ A_{4} \end{array}\right]\tilde{x}+\left[\begin{array}{c} A_{1}x^{0}\\ \begin{array}{c} f\left(x^{0}\right)\\ g\left(x^{0}\right) \end{array}\\ A_{3}x^{0}\\ \psi \left(x^{0}\right) \end{array}\right]=A\tilde{x}+L_{0},$$

#### 2.3.3. Optimal Estimation of the Parameters

There are various methods to determine the “best” parameter estimates. Maximum likelihood estimation yields the estimates for the unknown parameters which maximize the probability of obtaining the observations. If the probability density functions of both the measurement noise and unknown parameters are known, Bayesian estimation can be used. Another often-used method for stochastic estimation from noisy observations is Kalman filtering. The Kalman filter has been the subject of extensive research and application in the area of navigation. A Kalman filter does not need to store the measurement information during the entire observation period, and is suitable for real-time parameter estimation. For more information about Kalman filter, please refer to [21]. In this paper, the Kalman filter is used for parameter estimation.

During the element installation process, the element moves very slowly. The velocity is approximately 0.005 m/s. The tens of meters from the holding area to the final position in the trench takes between several and more than ten hours. In order to better monitor the possible lateral movement or rotation of the element, the integrated system is designed to measure at a frequency of 2 Hz. The parameter estimations of adjacent epochs are considered to be the same. In the prediction step of the Kalman filter, it is supposed that:

(17) X^k−=ΨX^k−1,

(18) $$P_{k}^{-}=\Psi P_{k-1}\Psi^{T}+Q,$$

therein, the superscript of minus represents the priori estimate, the subscript of *k* represents at the epoch *k*, X^k is the posteriori estimate of X at epoch k, Ψ relates the state at the previous time step k−1 to the state at the current step *k* and is unit matrix, $P_{k}$ is the posteriori estimate error covariance, and Q is the process noise covariance which is assumed to be constant.

The Kalman filter update is calculated using the following equations:

(19) $$K_{k}=P_{k}^{-}H^{T}{\left(HP_{k}^{-}H^{T}+R\right)}^{-1},$$

(20) X^k=X^k−+Kk(zk−HX^k−),

(21) $$P_{k}=\left(I-K_{k}H\right)P_{k}^{-},$$

where K is the Kalman gain, H=A, A is described in Section 2.3.2, R is the measurement noise covariance which is assumed to be constant, measurement vector $z_{k}=L$ and ***L*** is described in Section 2.3.2.

Q needs to be determined empirically. R is calculated from the observation error. The initial value of X^k−1 and $P_{k-1}$ are obtained by the least square adjustment.

### 2.4. Coordinate Calculation of Element Feature Points

**Figure 4:**
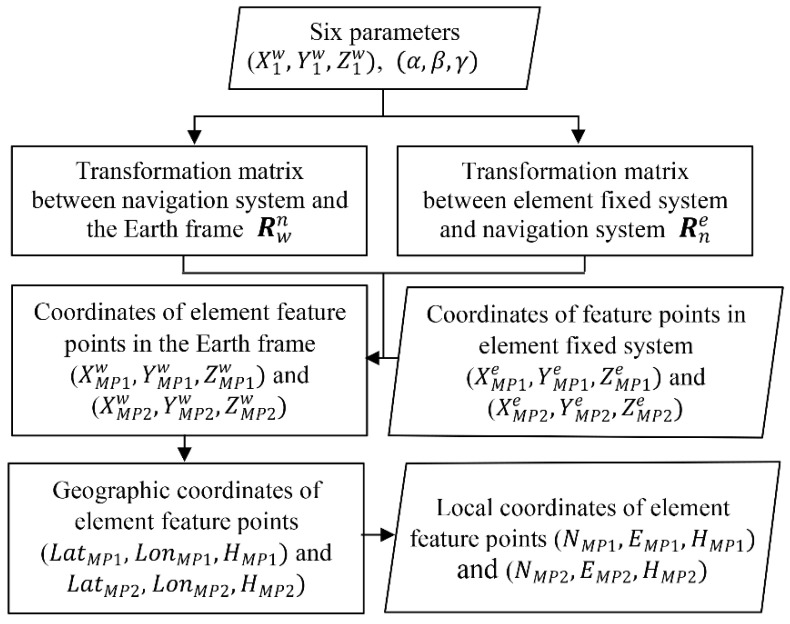
Flowchart of the coordinate calculation of element feature points.

After the accurate parameter vector X is determined, the coordinate calculation of element feature points should be conducted. The purpose is to calculate the local coordinates of MP1 and MP2. The element coordinates of MP1 and MP2, ($X_{MP1}^{e},Y_{MP1}^{e},Z_{MP1}^{e})$ and ($X_{MP2}^{e},Y_{MP2}^{e},Z_{MP2}^{e})$, are determined precisely before the element is launched. Figure 4 shows the process of coordinate calculation of MP1 and MP2.

Firstly, the transformation matrixes $R_{w}^{n}$ and $R_{n}^{e}$ are calculated. Then, the element fixed coordinates of MP1 and MP2 are transformed to WGS84 coordinates. Afterwards, the geodetic coordinates are converted to the geographic coordinates. Finally, according to [22,23], the geographic coordinates can be transformed to local plane coordinates, (N,E). In this paper, the local plane coordinates refer to Gauss-Krüger coordinates. The elevations are given as ellipsoidal heights. Hence, the local positions ($N_{MP1},E_{MP1},H_{MP1})$, ($N_{MP2},E_{MP2},H_{MP2})$ are determined.

## 3. Simulating Errors

The performance of the integrated positioning and attitude determination system is evaluated based on Monte Carlo simulations. To do this, the observation ***L*** should be simulated according to the given accuracy. In order to provide a generalized performance study, the observation errors should be set to be as close to reality as possible.

### 3.1. GNSS Errors

#### 3.1.1. GNSS Errors Distribution

In data processing, the observation is usually believed to obey normal distribution. In this case, the least square method can provide the minimum variance unbiased estimation of the parameters. For simulations, the GPS positioning and baseline estimation errors are usually assumed to be zero-mean Gaussian white noise. For example, [24] gives the baseline estimation error with respect to the baseline ratio, 1% to 10% and the error obeys the Gaussian distribution with zero mean.

However, [25] indicates that the observational error is not a normal distribution but a p-norm distribution because of the gross error in observation, model inaccuracy and other factors. p-norm distribution is symmetric and is a distributive class including Laplace, normal and rectangular distributions. The only assumption, that the error distribution is symmetric and has only one peak value, is needed if we use p-norm distribution to describe the errors [26]. Probability density function of p-norm distribution is as follows:

(22) $$f\left(x\right)=\frac{p\lambda }{2\sigma \Gamma \left(\frac{1}{p}\right)}exp\left\{-{\left[\lambda \frac{\left|x-\mu \right|}{\sigma }\right]}^{p}\right\},$$

where $\lambda ={\left[\frac{\Gamma \left(\frac{3}{p}\right)}{\Gamma \left(\frac{1}{p}\right)}\right]}^{1/2}$, Γ(x) is gamma function, μ and $\sigma^{2}$ are the mean and variance, respectively. Observation error is believed to obey p-norm distribution with p=1.4 after the measured GPS data are analyzed in [27]. In the following simulations, the GPS positioning and baseline estimation errors are assumed to be zero-mean p-norm distributed and p=1.4; the positional accuracy of antenna 1 is set as $\sigma_{N_{1}}=\sigma_{E_{1}}=0.02\;m$, $\sigma_{H_{1}}=1.5\sigma_{N_{1}}$; the baseline accuracy of baseline is $\sigma_{N_{12}}=\sigma_{E_{12}}=\sigma_{N_{13}}=\sigma_{E_{13}}=0.01\;m$, $\sigma_{H_{12}}=\sigma_{H_{13}}=1.5\sigma_{N_{12}}$.

Proportion of values of p-norm distribution and normal distribution within certain standard deviations are shown in Table 2 when μ=0, σ=1. Within the interval (μ−σ,μ+σ), the probability of p-norm distribution is slightly higher. Within the interval (μ−2σ,μ+2σ) and (μ−3σ,μ+3σ), the probability of normal distribution is slightly higher.

#### 3.1.2. Influence of Multipath Effects

**Figure 5:**
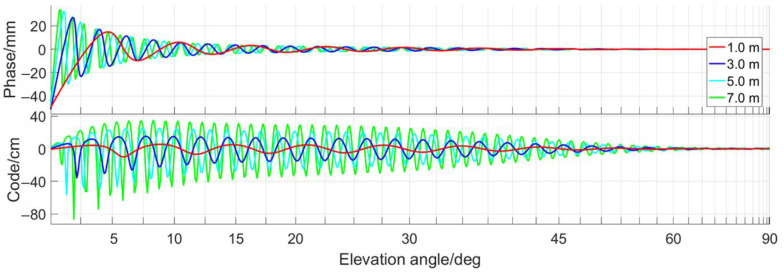
Effect of height of antenna above horizontal reflector on GNSS multipath errors.

Another factor to consider is the multipath effect. The GNSS multipath errors cannot be removed by differential methods since multipath is a localized phenomenon, which strongly depends on the environment of the measurement site, the performance of the antenna, and of the receiver. The GNSS antennas in the integrated positioning and attitude determination system of the immersed tunnel element are not far above the water surface. In the final stage of the element installation, the antennas may only be 2–5 m above the water. Multipath can be the major error contributor. As a first indicator to quantify the influence of multipath in this situation, the multipath simulator provided by [28] is used to examine the multipath effect on the GNSS code and carrier phase observations. A horizontal seawater surface with a negligible roughness, observed with a choke-ring antenna is assumed. Simulation results are shown in Figure 5. Changing the height of the antenna above the reflecting surface, as it will happen, while the tunnel element is immersed, changes the multipath error. Here error means the difference with respect to multipath-free conditions. The carrier phase multipath error which goes up to 40 mm for low elevation satellites, is at least an order of magnitude smaller than the pseudorange multipath error. When the elevation angle is greater than 15°, the carrier phase multipath error is less than 1 cm.

However, the influence of the multipath effect on the calculated position and baseline vector is still unclear. In order to further estimate the effect of multi-path errors on the resulting coordinates and baselines, we analyze the measured data of three GNSS points of HZM link immersed tunnel. The data were measured with R7 receivers (Trimble, Santa Clara, CA, USA) of which the static horizontal and vertical accuracy is 3 mm + 0.5 ppm and 5 mm + 0.5 ppm. Point CP01 is on an artificial island. Point CP02 and CP03 are on a measurement platform. CP02 and CP03 are about 5 m above the sea surface. The distance between CP01 and CP02 is about 400 m. The distance between CP02 and CP03 is about 6 m. The three points were observed for three consecutive days. The sampling interval is 15 s. RTKLIB (ver. 2.4.3) is used to process the observations [29].

**Figure 6:**
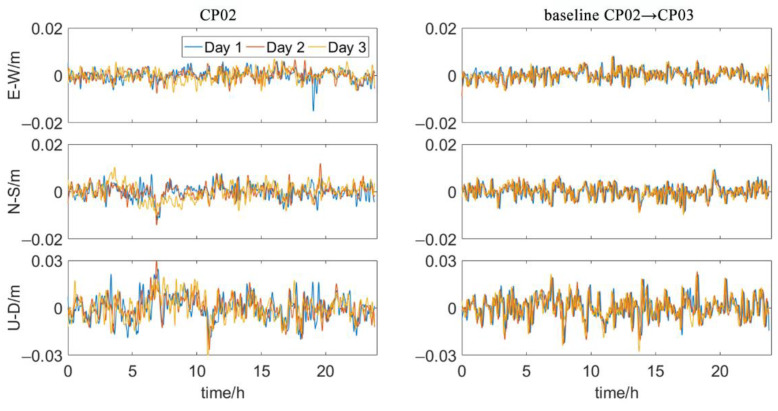
Coordinates and baseline sequences.

CP01 is regarded as the reference point and the coordinate sequence of CP02 is calculated in kinematic mode. Similarly, CP03 is used as the reference point and the baseline sequence of CP03→CP02 is calculated in kinematic mode. Due to the close distance between points, common errors such as ephemeris error, satellite clock error, ionospheric and tropospheric refraction error, and receiver related error can be effectively eliminated by differential processing. Hence, the position error and baseline error are mainly caused by the multipath effect, satellite visual condition and random noise. Figure 6 shows the coordinates and baseline sequences for three consecutive days. It can be seen that the point coordinates and baseline sequences have obvious repeatability, which accords with the characteristics of multi-path error. After deducting the random noise in the result, the influence of multipath error on coordinate and baseline in the east-west and north-south direction is less than 1 cm. The influence of multipath on coordinate and baseline in the up-down direction is less than 2 cm. Based on this investigation, we added additional 1cm, 1cm and 2cm multipath errors to the antenna coordinates and baseline observations in the respective east-west, north-south and up-down direction in the following analysis.

### 3.2. Roll and Pitch Angle Errors

The inclinometer used in the integrated system has two measuring axes. Install the inclinometer when the element is stable before the start of floating. The inclinometer should be installed carefully so that the inclinometer coordinate system is as parallel as possible to the element fixed coordinate system [30,31]. After an assembly error calibration, the inclinometer coordinate system is believed to be parallel to the element fixed coordinate system. During the immersion process, there will be no significant tilt of the element under the control of the immersion rig. The roll and pitch angle of the element can be measured directly.

Now, there are many kinds of high precision inclinometer with resolution better than 0.001° and precision better than 0.01°. Take a high precision inclinometer as an example, of which the root-mean-square error of multiple measurements (≥16) is 0.002°. It is reasonable that the accuracy of a single measurement can reach 0.015°. So, in the following simulations, it is assumed that the accuracy of the simulated roll and pitch is 0.015°, which conform to the zero-mean normal distribution.

### 3.3. Distance Errors

The distances are measured using an acoustic system. The distance error includes the systematic error caused by sound speed and random error. The sound velocity varies with temperature, salinity and pressure. The sound speed is described by the sound speed profile. Influenced by the sound velocity profile, the sound ray is a curve rather than a straight line between two points in water. Hence, the measured distance needs the correction of the curved ray [32]. If the sound speed profile is known, the sound ray tracing can be performed to obtain accurate curve sound ray correction. Now, there are multiple sound speed profile models, such as the Munk model [33], generalized digital environment model (GDEM) [34], empirical orthogonal function (EOF) [35] and layered sound speed profile model (LSSPM) [36]. LSSPM is adopted in this paper. For shallow seas less than 100 m deep, LSSPM is

(23) $$C\left(z\right)=a_{0}+a_{1}z+a_{2}z^{2},$$

Polynomial coefficients, $a_{0}$, $a_{1}$ and $a_{3}$, have different values in different sea areas and different seasons.

Assuming that the column of water through which the sound ray passes is evenly divided into *N* layers. The thickness of each layer, dz, is small enough that the speed of sound is considered constant within the layer. The horizontal distance of the beam within the layer is:

(24) $$y_{i}=dz*\tan\theta_{i},$$

**Figure 7:**
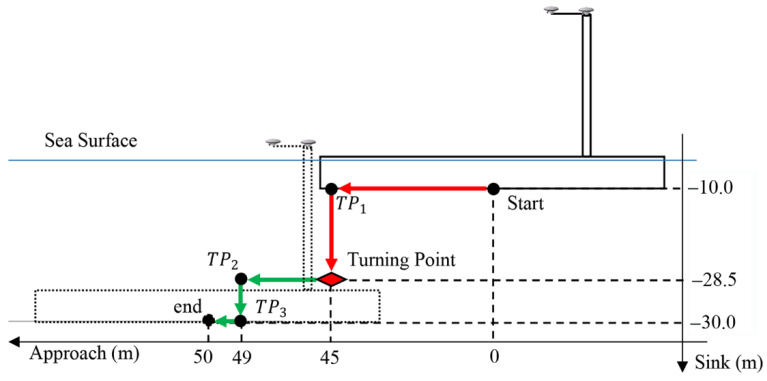
Element installation process. The red and green arrows indicate phase 1 and phase 2 of the process (side view, not proportional).

**Figure 8:**
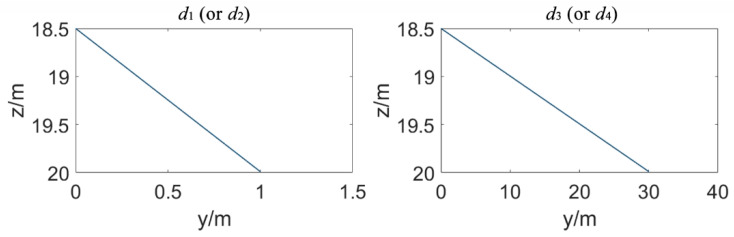
Sound ray tracing of $d_{1}$ (or $d_{2}$ ) and $d_{3}$ (or $d_{4}$ ). The sound rays traced are very close to straight lines. Changes in the speed of sound cause little bending of the sound rays.

The total horizontal distance of the beam is:

(25) $$y=\sum_{i=1}^{N}dz*\tan\theta_{i},$$

where $\;\theta_{i}$ is the refraction angle. When the coordinates of $P_{1}$–$P_{4}$ are known, all the refraction angles can be obtained using trial and error according to Equation (25), and then the true path of the ray can be traced. It is easy to know that the greater the height difference between the two elements and the closer the distances, the greater the correction of curved sound ray. The largest correction occurs when the secondary element is at $TP_{2}$, according Figure 7. The height of the element is assumed to be 10 m. The element installation path is shown in Figure 7. Polynomial coefficients, $a_{0}$, $a_{1}$ and $a_{3}$, are based on the recommended values for a particular sea area in [36]. The sound ray tracing of $d_{1}$ (or $d_{2}$) and $d_{3}$ (or $d_{4}$) is shown in Figure 8. The sound rays are very close to the straight lines. Changes in the speed of sound cause little bending of the sound rays. The maximum correction of curved sound ray is 0.008 m. For conservative considerations, the influence of a systematic error of 0.01 m in all simulated distance observations will be evaluated.

The random error of distance is assumed to be 1% of the distance [37,38]. At the same time, the random error is no less than 0.01 m. When 1% of the distance is less than 0.01 m, the random error is set to be 0.01 m.

## 4. Immersed Tunnel Element Immersion Simulation

### 4.1. Installation Scenario

The entire element installation process is shown in Figure 7, where for visualization purpose the actual data of a Hong Kong-Zhuhai-Macau link immersed tunnel element is used. The element moves down along a stairs-like route described by the red and green arrows for 70 m. The installation process is divided into two phases, named ‘phase 1′ (before the turning point) and ‘phase 2′ (after the turning point). Because of the relatively long distance, large height difference between the primary and secondary elements, and the relatively low accuracy requirement of the position and attitude during phase 1, there are no distance observations in phase 1. When the element reaches the turning point, the range measurement system starts to work.

In the simulation we assume, that the tunnel is along the east-west direction and that there is no slope in the vertical direction. The secondary element is on the east side of the primary one. Hence, the accuracy of the element in the north direction directly describes the axis accuracy of the tunnel. In this case, in order to keep the tunnel axis smooth, the north coordinates of MP1 and MP2 need to be accurately controlled. The accuracy requirement perpendicular to the axis and therefore in North coordinate is 3.5 cm during phase 2. The accuracy requirement is doubled to 7.0 cm during phase 1. The element size is 10 m×30 m×180 m. The distance between the measurement tower and the free end of the element is 30 m. The measurement tower is 30 m high. The two baselines are 5 m long. The angle between baseline 1→2 and 1→3 is 90°.

**Figure 9:**
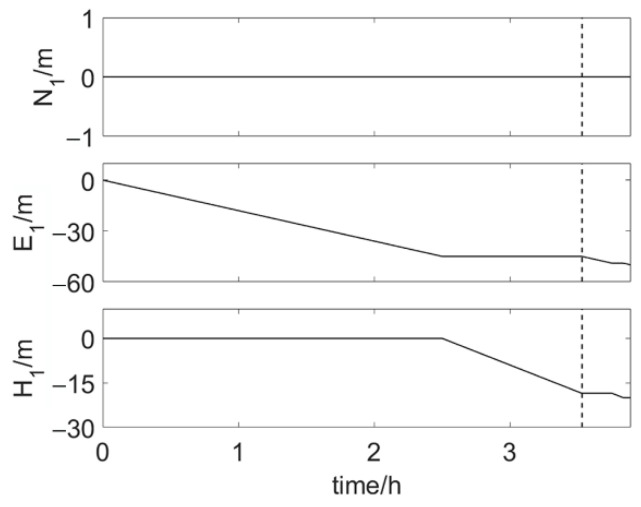
True position of antenna 1 relative to the initial moment during the whole element installation process. The vertical dotted line indicates the turning point.

The whole installation process of the element is simulated. The entire installation process takes 3.89 h, needing 28,000 epochs. In phase 1, there are 11 observations per epoch and it takes 3.53 h, needing 25,400 epochs; in phase 2, there are 15 observations per epoch and it takes 0.36 h, needing 2600 epochs. The true positions of antenna 1 relative to the initial moment during the whole element installation process are shown in Figure 9. In order to show the results of positioning and attitude determination more intuitively, the position of antenna 1 ($X_{1}^{w},Y_{1}^{w},Z_{1}^{w})$ is transformed to the local coordinate ($N_{1},E_{1},H_{1})$.

### 4.2. Observation Error Scenarios

The observations may be affected by different error sources during the element installation process. In order to assess the influence of GNSS multipath error and the distance systematic error on coordinates of MP1 and MP2 in phase 2, the observations with different errors are simulated. The multipath effect on coordinate of antenna 1, baseline 1→2 and 1→3 in the east-west and north-south direction is set to be 1 cm. The multipath error in the up-down direction is set to be 2 cm. GNSS multipath error lasts for 90 s. The four distances of all epochs are assumed to contain the systematic error of 0.01 m. The observation error scenarios include:

(1) Error scenario 1: only zero mean random errors, no multipath errors or distance systematic errors;
(2) Error scenario 2: multipath error in coordinates of antenna 1, baseline 1→2 and 1→3 simultaneously in phase 2 (horizontal 1 cm, vertical 2 cm);
(3) Error scenario 3: multipath error in baseline 1→3 in phase 2 (horizontal 1 cm, vertical 2 cm);
(4) Error scenario 4: systematic distance errors in phase 2 (1 cm), no multipath errors.

### 4.3. Results and Discussion

For each error scenario, 50,000 simulations were performed and the results were similar. In the following, we only show the results of one simulation for each error scenario. The true values of parameters and coordinates of element feature points in the simulation are known, so the accuracy of the system is expressed by the true error.

#### 4.3.1. Error Scenario 1

**Figure 10:**
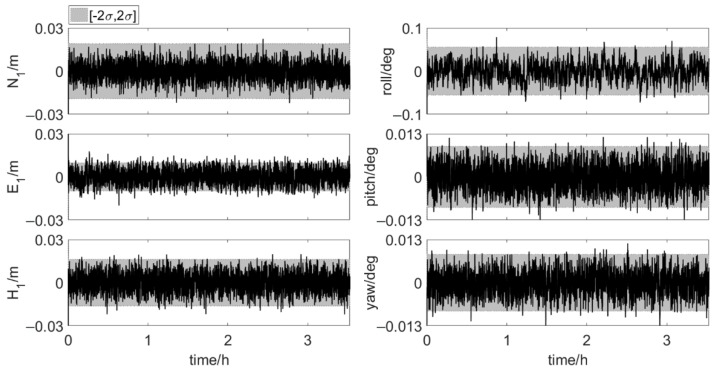
True errors of six parameters, shaded area: ±2*σ* set 1: phase 1, error scenario 1).

**Figure 11:**
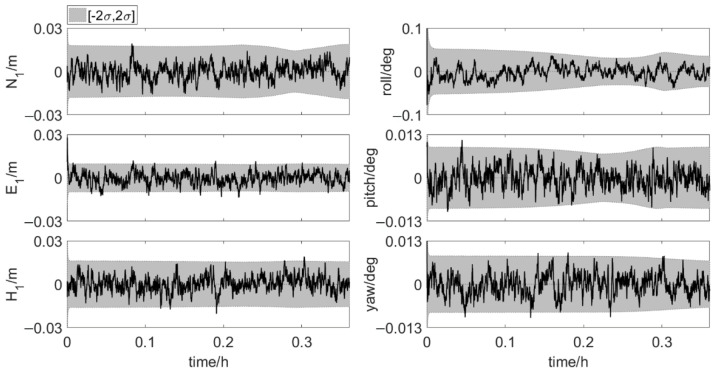
True errors of six parameters, shaded area: ±2σ (set 2: phase 2, error scenario 1).

Figure 10 and Figure 11 show the results of six parameters during phase 1 and phase 2 of the installation process. σ is the square root of the diagonal elements of the error covariance matrix during Kalman filtering, which is called error. Errors of position of antenna 1 and three attitude angles vary significantly during phase 2. This is because as the relative position of the two elements changes, the accuracy of distances changes. Errors of the six parameters are shown in Table 3. For the position of antenna 1, error of $E_{1}$ is less than error of $N_{1}$ and $U_{1}$. The order of attitude angles error from small to large is yaw error, pitch error and roll error. Most of the true errors fall within the range of [−2σ, 2σ].

**Figure 12:**
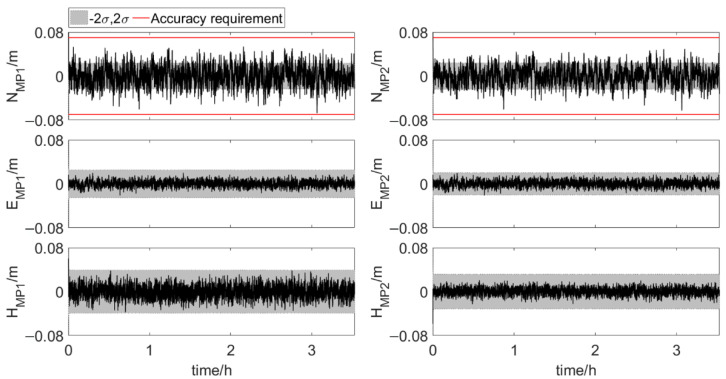
True errors of element feature points positions, shaded area: ±2σ (set 1: phase 1, error scenario 1).

**Figure 13:**
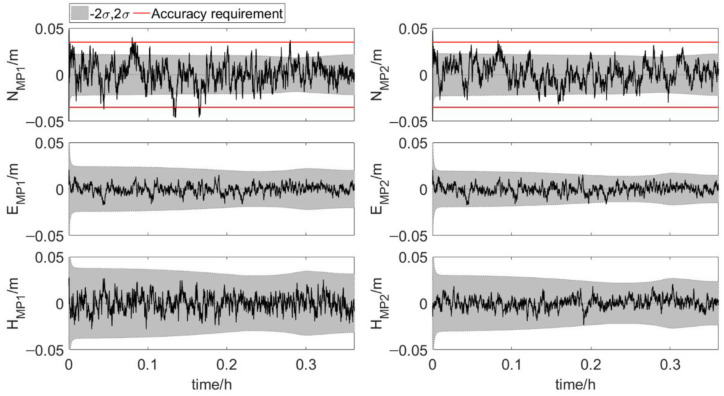
True errors of element feature points positions, shaded area: ±2σ (set 2: phase 2, error scenario 1).

Figure 12 and Figure 13 show the results of the element feature points positions during phase 1 and phase 2. The position error, σ, is calculated according to the error propagation law. The position errors of MP1 and MP2 vary significantly during phase 2, which is due to the change of the errors of antenna 1 position and three attitude angles. The position errors of the feature points in the last part of phase 2 are shown in Table 3. Both for MP1 and MP2, the order of coordinate component error from small to large is east-west error, north-south error and up-down error. And as shown in Table 4, 99.9% of the north true errors of MP1 and MP2 are within the accuracy requirement. More than 80% of the north true errors of MP1 and MP2 are within the range of [−2σ, 2σ]. When the two elements are very close, the true errors of the feature points position are less than 0.02 m in the directions perpendicular to and along the tunnel axis. It indicates that the integrated system has high positioning accuracy. The integrated system, which uses Kalman filter for dynamic estimation of parameters, is suitable for real-time scenarios. Hence, the integrated positioning and attitude determination system can provide the absolute position of the element with sufficient accuracy in real time during the element immersion process.

#### 4.3.2. Error Scenario 2 and 3

**Figure 14:**
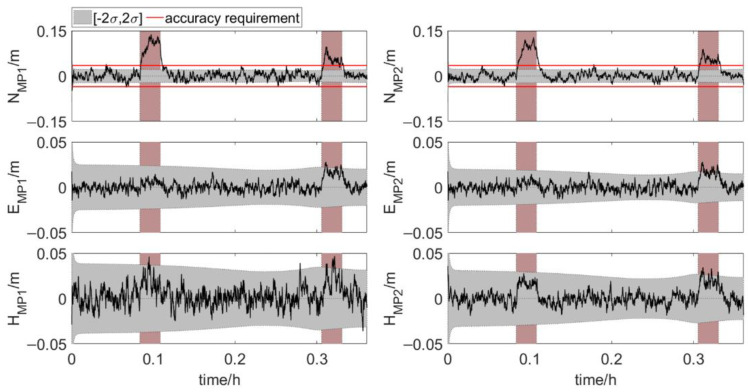
True errors of element feature points positions. The rosy region is the time period with multipath errors, shaded area: ±2σ (error scenario 2).

When the coordinates of antenna 1, baseline 1→2 and 1→3 contain multipath errors simultaneously, the element feature points positions during phase 2 are shown in Figure 14. The north true errors of MP1 and MP2 show obvious fluctuations during the time period with multipath errors and are far beyond the accuracy requirement. With the addition of multipath errors, large deviations of north true error quickly appear, and then the deviations gradually decrease. The north true errors fluctuate more during the first period of multipath error. This is because the accuracy of the distances in this interval is lower than the accuracy of the distances in the second multipath error period. The higher the accuracy of the distances, the smaller the influence of multipath error of the same size. The east and vertical true error sequences also have a certain degree of deviation. The deviations are not too large, about 2σ.

**Figure 15:**
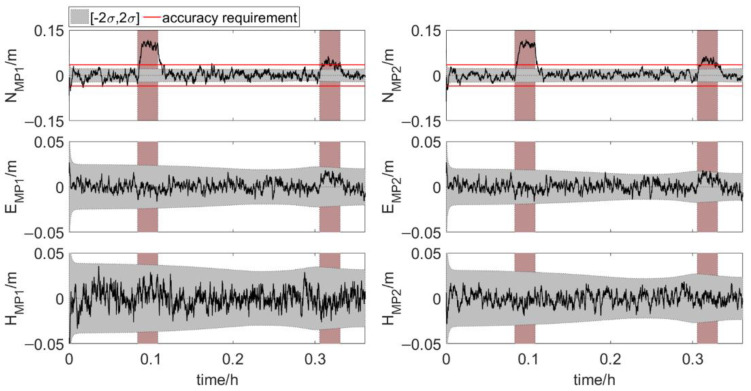
True errors of element feature points positions. The rosy region is the time period with multipath errors, shaded area: ±2σ (error scenario 3).

The multipath errors in the coordinates of antenna 1 and baseline 1→2 have no obvious effects on the element positions. The large fluctuations of true error sequences in Figure 14 are mainly caused by the multipath errors in baseline 1→3. Figure 15 shows the element feature points positions during phase 2 when only baseline 1→3 contains multipath error. The north true errors of the two points show fluctuations similar to those shown in Figure 14 during the time period with multipath errors. The east and vertical true errors are similar to those shown in Figure 13. 

**Figure 16:**
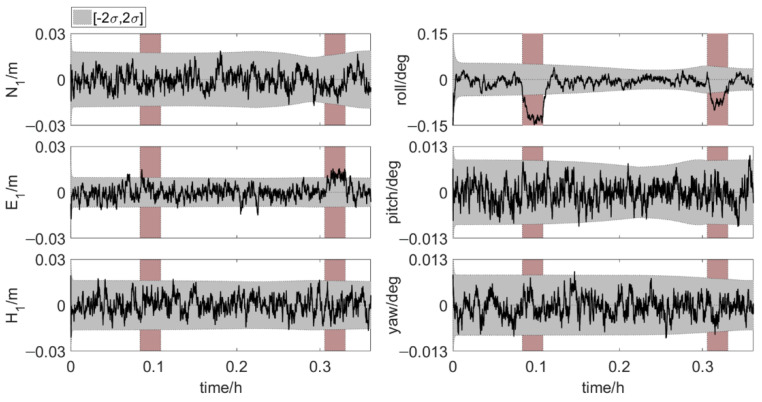
True errors of six parameters. The rosy region is the time period with multipath errors, shaded area: ±2σ (error scenario 3).

The multipath errors in baseline 1→3 have much more obvious effect on the element positions than the multipath errors in the coordinates of antenna 1 and baseline 1→2. This is mainly because the multipath errors in baseline 1→3 cause a large deviation in the roll angle, as shown in Figure 16.

The multipath error in baseline 1→3 has the greatest effects on the integrated system. It can cause the lateral positioning accuracy of element to miss the accuracy requirement. The multipath error, especially the error in baseline 1→3, cannot be ignored. Measures need to be taken to reduce its impact. But this is beyond the scope of this paper.

#### 4.3.3. Error Scenario 4

**Figure 17:**
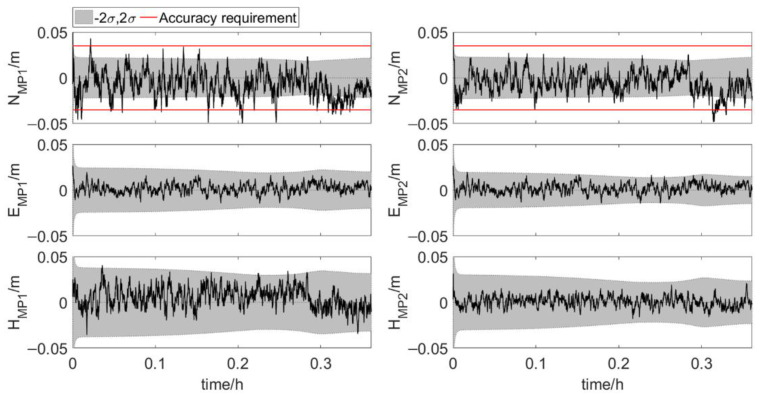
True error of element feature points positions, shaded area: ±2σ (error scenario 4).

The feature points positions in phase 2 are shown in Figure 17 when the four distances of all epochs contain the systematic error of 0.01 m. There is some systematic deviation in the north true errors, especially in the last part. Because when the two elements are very close, the random error of the distance is very small, and the systematic error of 0.01 m makes the relative accuracy of the distance decay significantly. However, most of the north true errors of MP1 and MP2 are still within the accuracy requirement. The east and vertical components of the two points results are similar to those shown in Figure 13. The distance systematic error only has slight effects on the system. But in practical application, it is still necessary to take measures to avoid the systematic error of distances.

## 5. Conclusions

In this paper, a newly-developed integrated positioning and attitude determination system has been presented, which is intended to provide high-accurate element position and attitude in real time during element immersion process, especially in the final stage. The integrated system consists of a measurement tower with three GNSS antennas, an inclinometer and a range measurement system. The performance of the integrated positioning and attitude determination system is evaluated based on Monte Carlo simulations. 99.9% of the element positions in the direction perpendicular to the tunnel axis meet the accuracy requirement. The GNSS multipath errors in the coordinates of antenna 1 and in the baseline along the longitudinal direction of the element have no obvious effects on the element positions. The GNSS multipath errors in the baseline along the transverse direction of the element cause the element position to show obvious fluctuations in the direction perpendicular to the tunnel axis during the time period with multipath errors. The distance systematic error only has slight effects on the element position in the direction perpendicular to the tunnel axis.

As a final conclusion, if there is no multipath error, the newly-developed integrated positioning and attitude determination system can provide high-accuracy absolute positions of both ends and attitude angles of the element during the entire installation process in real time with just one measurement tower. Further consideration should be given to mitigating the impact of GNSS multipath errors, especially the errors in the baseline along the transverse direction of the element.

## Figures and Tables

**Table 1:** The four coordinate systems used.

Coordinate System	Definition
ECEF Coordinate System	Origin: the earth’s center of mass; *Z*-axis: the earth’s rotation axis defined by the Conventional Terrestrial Pole; *X*-axis: the intersection of the orthogonal plane to *Z*-axis and Greenwich mean meridian; *Y*-axis: orthogonal to *X*-axis and *Z*-axis, making the system directly oriented.
Navigation coordinate system	It is formed from a plane tangent to the Earth’s surface. The three axes point east, north and perpendicular to the tangent plane and away from the center of the earth.
Element-fixed coordinated system	It is defined by the plane of the upper surface of the element. See in Figure 3.
Local plane coordinate and height system	It is realized by Gauss–Krüger or Universal Transverse Mercator projection. The elevations are given as ellipsoidal heights.

**Table 2:** Proportion of values within certain standard deviations, μ=0, σ=1, *p* = 1.4.

Distribution	μ−σ<x<μ+σ	μ−2σ<x<μ+2σ	μ−3σ<x<μ+3σ
Normal distribution	68.27%	95.45%	99.73%
p-norm distribution	71.89%	94.53%	99.19%

**Table 3:** Errors of six parameters and position of element feature points (set 1 and set 2).

σ (m or °)	Phase 1	Last Part of Phase 2	σ (m)	Phase 1	Last Part of Phase 2
$N_{1}$	0.010	0.009	$N_{MP1}$	0.012	0.011
$E_{1}$	0.005	0.005	$E_{MP1}$	0.013	0.010
$H_{1}$	0.010	0.008	$H_{MP1}$	0.020	0.016
α	0.028	0.018	$N_{MP2}$	0.012	0.011
β	0.005	0.005	$E_{MP2}$	0.010	0.007
γ	0.005	0.004	$H_{MP2}$	0.016	0.012

**Table 4:** Probability of feature points in north within threshold and the error range of [−2σ, 2σ] (set 1 and set 2).

Element Feature Points	Phase 1		Phase 2	
	Within Accuracy Requirement	Within [−2σ, 2σ]	Within Accuracy Requirement	Within [−2σ, 2σ]
MP1	99.9%	84.6%	99.9%	85.2%
MP2	99.9%	90.4%	99.9%	90.0%
